# Machine Learning Models for the Prediction of Preterm Birth at Mid-Gestation Using Individual Characteristics and Biophysical Markers: A Cohort Study

**DOI:** 10.3390/children12111451

**Published:** 2025-10-25

**Authors:** Antonios Siargkas, Ioannis Tsakiridis, Dimitra Kappou, Apostolos Mamopoulos, Ioannis Papastefanou, Themistoklis Dagklis

**Affiliations:** 1 Third Department of Obstetrics and Gynecology, School of Medicine, Faculty of Health Sciences, Aristotle University of Thessaloniki, 54124 Thessaloniki, Greece; asiargk@auth.gr (A.S.); amamop@auth.gr (A.M.); dagklis@auth.gr (T.D.); 2 Institute of Life, IASO General Hospital, 15123 Athens, Greece; 3 Fetal Medicine Clinic, Monis Petraki 4, Kolonaki, 11521 Athens, Greece; 4 Department of Women and Children’s Health, Faculty of Life Sciences and Medicine, King’s College London, London WC2R 2LS, UK

**Keywords:** iatrogenic preterm birth, interpretable models, machine learning, prediction, preterm birth, risk stratification, second trimester screening, spontaneous preterm birth

## Abstract

**Highlights:**

**What are the main findings?**

- Predictive models demonstrated significantly stronger predictive performance for iatrogenic preterm birth (PTB) compared to spontaneous PTB across all gestational age thresholds. Traditional Logistic Regression performed comparably to more complex machine learning algorithms, indicating that predictor selection and subtype stratification are more critical for performance than algorithmic complexity.
- The predictive accuracy of the models consistently improved for earlier, more severe degrees of prematurity for both subtypes. For instance, the top AUC for predicting iatrogenic PTB increased from 0.764 at <37 weeks to 0.862 at <32 weeks.

**What is the implication of the main finding?**

- The development of PTB subtype-specific models allows for a more personalized risk assessment than using single risk factors, which is crucial as management strategies differ substantially for spontaneous and iatrogenic PTB. A high predicted risk of spontaneous PTB might lead to progesterone therapy, while a high risk for iatrogenic PTB would prompt intensified surveillance for conditions like pre-eclampsia and fetal growth restriction.
- Accurate risk stratification enables the timely administration of interventions like antenatal corticosteroids and facilitates logistical planning for neonatal intensive care resources and potential transfers to specialized centers.

**Abstract:**

Background/Objectives: Preterm birth (PTB), defined as birth before 37 completed weeks of gestation, is a major global health challenge and a leading cause of neonatal mortality. PTB is broadly classified into spontaneous and medically indicated (iatrogenic), which have distinct etiologies. While prediction is key to improving outcomes, there is a lack of models that specifically differentiate between spontaneous and iatrogenic PTB subtypes. This study aimed to develop and validate predictive models for the prediction of spontaneous and iatrogenic PTB at <32, <34, and <37 weeks’ gestation using medical history and readily available second-trimester data. Methods: This was a retrospective cohort study on singleton pregnancies from a single tertiary institution (2012–2025). Predictor variables included maternal characteristics, obstetric history, and second-trimester ultrasound markers. Four algorithms, including multivariable Logistic Regression and three machine learning methods (Random Forest, XGBoost, and a Neural Network), were trained and evaluated on a held-out test set (20% of the data). Model performance was primarily assessed by the Area Under the Curve (AUC). Results: In total, 9805 singleton pregnancies were included. The models performed significantly better for iatrogenic PTB than for spontaneous PTB. For delivery <37 weeks, the highest AUC for iatrogenic PTB was 0.764 (Random Forest), while for spontaneous PTB it was 0.609 (Neural Network). Predictive accuracy improved for earlier gestations; for delivery <32 weeks, the best model for iatrogenic PTB achieved an AUC of 0.862 (Neural Network), and the best model for spontaneous PTB achieved an AUC of 0.749 (Random Forest). Model interpretation revealed that iatrogenic PTB was primarily driven by markers of placental dysfunction, such as estimated fetal weight by ultrasound scan and uterine artery pulsatility index, while spontaneous PTB was most associated with a history of PTB and a short cervical length. Conclusions: Models using routine mid-gestation data demonstrate effective prediction for iatrogenic PTB, with accuracy improving for earlier, more severe cases. In contrast, performance for spontaneous PTB was modest. Traditional Logistic Regression performed comparably to complex machine learning algorithms, highlighting that the clinical value is rooted in the subtype-specific modeling approach rather than in algorithmic complexity.

## 1. Introduction

Preterm birth (PTB), defined by the World Health Organization as delivery occurring before 37 completed weeks of gestation [1], complicates about 10% of the obstetric population and represents a major global health challenge [2]. The etiology of PTB is broadly classified into two subtypes: spontaneous onset, which encompasses 75–80% of cases arising from either preterm labor or preterm prelabor rupture of membranes, and medically indicated birth, which accounts for the remaining 20–25% and results from obstetric intervention [3]. The global incidence of PTB has shown a concerning increase over the last years [4]. The profound clinical impact of prematurity is highlighted by its status as the leading cause of neonatal mortality worldwide, responsible for over one million infant deaths in a year and significant lifelong morbidities among survivors [5]. In particular, the consequences of PTB include a wide spectrum of severe short- and long-term health complications; immediate neonatal morbidities frequently include respiratory distress syndrome, intraventricular hemorrhage, bronchopulmonary dysplasia, necrotizing enterocolitis, retinopathy of prematurity, periventricular leukomalacia, and sepsis [6,7,8]. For infants who survive these initial challenges, the long-term sequelae can be debilitating, often manifesting as cerebral palsy or other significant cognitive and neurobehavioral deficits [9,10]. Beyond the direct health impact on the individual and their family, prematurity imposes a substantial socioeconomic burden. The costs associated with prolonged stays in neonatal intensive care units, recurrent hospitalizations, and the need for specialized long-term follow-up are considerable [11].

A central challenge in PTB prediction and prevention is its complex and multifactorial etiology. Major risk factors for spontaneous PTB are a prior preterm delivery or a short cervix and multivariable predictive models that integrate these key indicators with other clinical and obstetric characteristics yield superior prognostic value compared to standalone risk factors [12]. Effective risk stratification for spontaneous PTB in the general obstetric population is of considerable clinical importance, as it facilitates the timely application of prophylactic measures for pregnancies identified as high risk [13]. In pregnancies at high risk for spontaneous PTB, the primary therapeutic options that have been systematically evaluated include hormonal support with vaginal progesterone, surgical reinforcement via cervical cerclage, and mechanical support with a silicone pessary [14,15]. Iatrogenic PTB, on the other hand, is mainly the result of placental dysfunction in the form of preeclampsia (PE), fetal growth restriction (FGR) and stillbirth. Recent data suggest that early aspirin administration in women at high risk for preterm PE may reduce the rate of preterm deliveries in pregnancies with clinical manifestations of placental dysfunction and the overall severity of PTB [13].

Accurate prediction is the key to optimizing perinatal outcomes in both spontaneous and iatrogenic PTB, by allowing for the timely administration of interventions including antenatal corticosteroids and magnesium sulfate [16,17]. It also offers significant logistical benefits, enabling better planning of neonatal intensive care resources and transfer to specialized centers [10,18]. The second trimester is widely regarded as a crucial stage for PTB risk assessment. This timeframe offers a balance between achieving greater predictive accuracy and allowing sufficient opportunity for implementing prophylactic measures and stratifying our population into different intensities of care [12,19,20]. To date, one of the most powerful second-trimester models for PTB achieved an Area Under the Curve (AUC) of 0.75; its performance relied on incorporating numerous socioeconomic variables alongside standard medical data, which are not easily accessible, and combined both spontaneous and iatrogenic PTB [21]. Other relevant studies that utilized more restricted, clinically focused variable sets from the second trimester have reported models with AUC scores below 0.75 [22]. Furthermore, the prediction of spontaneous PTB has proven particularly challenging, with current models demonstrating limited accuracy with data from both the first [23] and the second trimester [24], a fact often attributed to the multifactorial pathophysiology of this condition [25]. Conversely, while the clinical pathways leading to iatrogenic PTB are often more defined, this subtype has been largely overlooked in prediction research, with a significant scarcity of dedicated models [26]. Interestingly, we found only one study that investigated prediction in iatrogenic PTB, and it was limited to women with scarred uteruses [27]. This clear gap in the literature, compounded by methodological limitations in many existing studies, underscores the need for robust, subtype-specific models that can be readily translated into clinical practice. Crucially, by treating PTB as a single outcome, previous models may have been limited, as predictors for spontaneous and iatrogenic subtypes can have different or even opposing effects, potentially obscuring important predictive signals.

Therefore, the objective of this study was to develop and internally validate robust predictive models for spontaneous and iatrogenic PTB at <32, <34, and <37 weeks’ gestation, comparing the performance of traditional Logistic Regression with several machine learning algorithms. Our approach utilized a set of readily available and cost-effective variables derived from maternal history and routine second-trimester ultrasound examinations, aiming to provide a practical and effective tool for risk stratification in a contemporary antenatal care setting.

## 2. Materials and Methods

### 2.1. Study Design and Setting

This retrospective cohort study included a consecutive sample of women who attended the 3rd Department of Obstetrics and Gynecology, School of Medicine, Faculty of Health Sciences, Aristotle University of Thessaloniki, Greece, between April 2012 and May 2025. All participants received routine antenatal care within this single tertiary institution, and all ultrasound examinations were performed by fetal medicine specialists accredited by the Fetal Medicine Foundation, London, UK. Data for this study were extracted from dedicated electronic medical records, which encompassed detailed demographic, clinical, and sonographic information.

### 2.2. Ethical Considerations and Reporting Standards

Informed consent was obtained from all participants, which included permission for their anonymized data to be used for possible future research purposes. As the study involved the retrospective analysis of routinely collected clinical data with no patient intervention, a formal review by an institutional board was not required. The reporting of this observational study adhered to the principles outlined in the Strengthening the Reporting of Observational Studies in Epidemiology (STROBE) guidelines [28]. Furthermore, the development and reporting of the analytical model followed the Transparent Reporting of a Multivariable Prediction Model for Individual Prognosis or Diagnosis (TRIPOD) recommendations [29].

### 2.3. Study Population and Data Collection

The study population comprised all women with viable singleton pregnancies who underwent both a first-(11^+0^–13^+6^ weeks) and second-trimester (20^+0^ and 23^+6^ weeks) ultrasound scan at our department. Exclusion criteria included multiple gestations, pregnancies with known fetal genetic or major structural anomalies, termination of pregnancy, and miscarriage before 23^+6^ weeks. A standardized protocol was used to systematically collect clinical data, including maternal demographics, anthropometrics, obstetric history, lifestyle factors, and pre-existing medical conditions, which were recorded in the Astraia electronic database.

### 2.4. Fetal Medicine Assessment

All second-trimester sonographic assessments were performed by Fetal Medicine Foundation certified sonographers, ensuring high consistency in examination protocols and measurement techniques. These examinations involved a standardized fetal anatomy survey and biometry. Fetal head circumference, abdominal circumference, and femur length were measured to calculate an estimated fetal weight (EFW) using the Hadlock formula [30]. Transabdominal uterine artery (UtA) Doppler velocimetry was conducted according to the International Society of Ultrasound in Obstetrics and Gynecology (ISUOG) guidelines [31]. The pulsatility index (PI) was measured for both the left and right uterine arteries, and the average of these two values was used for analysis. Gestational age was defined based on the crown–rump length measured during the 11–13 weeks’ scan.

### 2.5. Investigated Outcomes

This study investigated the prediction of PTB, which was stratified into its two primary clinical subtypes: spontaneous and iatrogenic. Spontaneous PTB was defined as delivery before 37 weeks of gestation resulting from either spontaneous preterm labor or preterm prelabor rupture of membranes. Iatrogenic PTB was defined as a medically indicated delivery before 37 weeks of gestation, accomplished via induction of labor or a planned cesarean section. To evaluate model performance across varying degrees of prematurity, each subtype was further analyzed at three distinct gestational age thresholds: delivery at <37, <34, and <32 completed weeks. This framework resulted in six discrete binary outcomes, for which independent predictive models were developed and assessed.

### 2.6. Statistical Analysis

The initial analysis involved a comprehensive exploratory phase. The distributions of all continuous predictor variables were visually assessed using histograms and density plots to determine their normality. Subsequently, a baseline characteristics table was generated to summarize and compare the study population, stratified by delivery outcome (PTB vs. term delivery). For this comparison, normally distributed continuous variables were presented as mean (standard deviation) and compared using Student’s *t*-test, while non-normally distributed variables were presented as median [interquartile range] and compared using the Mann–Whitney U test. Categorical variables were presented as frequencies and percentages and were compared using the Chi-squared test or Fisher’s exact test, as appropriate.

### 2.7. Predictor Variables and Modeling Strategy

In our models, we included three types of data, based on clinical significance and availability for second trimester prediction of PTB:

- Maternal characteristics and history: Maternal age, height, pre-pregnancy weight, use of assisted reproductive technology, smoking status, first trimester bleeding, presence of a cervical cerclage, previous cesarean section, pre-existing diabetes, chronic hypertension, thrombophilia, thyroid disorder, a prior loop electrosurgical excision procedure or a large loop excision of the transformation zone procedure, a history of preterm delivery, and multiparity.
- Ultrasound markers: Gestational age at examination determined by CRL, estimated fetal weight (EFW), mean uterine artery pulsatility index (UtA-PI), suspected vasa previa, and cervical length.

### 2.8. Feature Selection and Model Development

The following multi-stage process was performed independently for every outcome. A robust, data-driven feature selection process was conducted only on the maternal characteristics and history variables to identify the most predictive and parsimonious core set of predictors for each outcome. This process involved the following:

- Variable importance ranking: A Random Forest model was trained on all available maternal history predictors using 5-fold cross-validation and down-sampling to generate a stable, ranked list of variables based on their predictive importance.
- Iterative subset evaluation: The algorithm then iteratively built and tested new Random Forest models on incrementally larger subsets of the top-ranked predictors, specifically testing subsets. The cross-validated AUC was recorded for each subset size.
- Parsimonious selection: The iterative evaluation revealed that the model using the complete set of maternal history variables achieved the highest AUC score. The analysis showed that no smaller, more parsimonious subset of features achieved a performance within 0.5 standard deviations of this top score. Therefore, to maximize the information available to the models, the decision was made to retain all maternal history and characteristic variables for the final model development.

After this exploratory analysis confirmed the utility of the full maternal dataset, the ultrasound markers were added to the model.

Dataset preparation and partitioning: For each outcome, the dataset containing the final selected features was prepared. To handle missing data points, k-Nearest Neighbors (k-NN) imputation (k = 5) was employed. This method was chosen to preserve inter-variable relationships by using the full predictor set to inform its estimates. The complete dataset was then partitioned into a training set (80%) and a testing set (20%) using stratified sampling to maintain the outcome distribution in both sets.

### 2.9. Model Training and Validation

A rigorous internal validation process was conducted on the training set using 10-fold cross-validation repeated 3 times. Within this framework, several key steps were performed:

1. Data pre-processing: Prior to fitting each model, the continuous predictor variables were centered and scaled. This standardization process ensures that all variables are on a comparable scale.
2. Class imbalance correction: To address the significant class imbalance inherent in predicting PTB, random undersampling was employed within the cross-validation process. This technique prevented the models from developing a bias towards predicting the majority (term) class by training on a balanced subset of data [25].
3. Algorithm training: Following these steps, four distinct classification algorithms were trained:
  ○ Multivariable Logistic Regression: A standard Logistic Regression model was fitted using the main effects of the selected predictors.
  ○ Random Forest: Models were trained with 500 trees, and the number of variables sampled at each split (mtry) was tuned via the cross-validation process.
  ○ eXtreme Gradient Boosting (XGBoost): An automated tuning process optimized a suite of key hyperparameters, including the number of boosting rounds, maximum tree depth, and learning rate.
  ○ Single-hidden-layer Artificial Neural Network: The network’s key hyperparameters, the number of neurons in the hidden layer (size) and the weight decay regularization parameter (decay), were tuned via cross-validation.

By applying these steps inside the cross-validation loops, we ensured that hyperparameter tuning and model training were performed robustly, leading to an unbiased estimate of performance on the final held-out test set.

### 2.10. Model Performance Evaluation

The final, trained models were evaluated on the held-out testing set. Model performance was primarily assessed by the AUC with its 95% Confidence Intervals (CI). For the <37 weeks outcome, the statistical significance of the difference in AUC for the logistic regression and the Random Forest model between the spontaneous and iatrogenic outcomes was evaluated using DeLong’s test. Additional performance metrics calculated included accuracy, sensitivity, specificity, Positive Predictive Value (PPV), and the F1-Score. Performance was also assessed by calculating the sensitivity of each model at a fixed specificity of 80%.

### 2.11. Model Interpretation

To enhance model interpretability, the final logistic regression and Random Forest models for the <37 weeks outcome were further analyzed using SHapley Additive exPlanations (SHAP) values [32]. This technique, originating from cooperative game theory, provides a method to fairly attribute the output of a prediction among the different input features by quantifying the marginal contribution of each predictor to the model’s decision for each individual case. The analysis was implemented in R, primarily utilizing the DALEX package to create model-agnostic explainers for the final, trained models using the held-out test data. Subsequently, the predict parts function was employed to compute the SHAP values for every prediction within the test set.

The resulting SHAP summary plots aggregate these instance-level values to provide a global understanding of feature importance and impact. In these plots, features are ranked vertically by their overall importance, which is calculated as the mean absolute SHAP value across all test set instances. The length of the horizontal bar for each feature represents the average magnitude of its impact on the model’s output probability. The color of the bar indicates the direction of the effect: green bars represent a positive contribution that increases the predicted risk of PTB, while red bars represent a negative contribution that decreases the predicted risk. Finally, the variability of each feature’s impact across the dataset is represented by the interquartile range of its SHAP values, shown as a dark line overlaying each bar.

All statistical and ML analyses were conducted using R software (Version 4.3.2 or later), with a fixed random seed set for reproducibility. The primary analysis relied on the caret package for its unified modeling framework, with specific models implemented via Random Forest, xgboost, and nnet. Additional key packages included dplyr for data manipulation, pROC for receiver operating characteristic (ROC) curve analysis, tableone for descriptive statistics, and ggplot2 for data visualization.

## 3. Results

### 3.1. Study Population

A total of 9805 pregnancies met the inclusion criteria and were included in the final analysis. Of these, 857 (8.7%) resulted in PTB. The baseline characteristics of the study population, stratified by the PTB < 37 weeks outcome, are detailed in Table 1.

### 3.2. Model Performance

The feature selection process resulted in the retention of all maternal history and characteristic variables. These variables, supplemented by key second-trimester ultrasound data, constituted the final set of predictors used to develop the models. For detailed analysis, two primary models were selected: the Random Forest model, due to its generally superior predictive performance, and the logistic regression model, chosen for its widespread application and value in clinical interpretation.

### 3.3. Prediction of Spontaneous and Iatrogenic PTB < 37 Weeks

For the prediction of spontaneous PTB < 37 weeks, the models showed modest performance. The Neural Network model achieved the AUC at 0.609 (95% CI 0.546–0.671). At its optimal threshold, this model had a sensitivity of 0.430 and a specificity of 0.700. When calibrated to a fixed specificity of 80%, the Random Forest model achieved the highest sensitivity of 36.0% (Table 2).

The models demonstrated significantly stronger performance for predicting iatrogenic PTB < 37 weeks. The Random Forest model was the top performer with an AUC of 0.764 (95% CI 0.710–0.818), closely followed by the Neural Network (AUC: 0.761) and logistic regression (AUC: 0.760). At its optimal threshold, the Logistic Regression model yielded a sensitivity of 0.675 and a specificity of 0.724. At a fixed specificity of 80%, the Logistic Regression model maintained a sensitivity of 60.0% (Table 3).

### 3.4. Prediction of Spontaneous and Iatrogenic PTB < 34 Weeks

In predicting spontaneous PTB < 34 weeks, the Random Forest model achieved the highest AUC of 0.678 (95% CI 0.525–0.831). The Logistic Regression model, however, had the highest sensitivity of 0.579 at its optimal threshold. At a fixed 80% specificity, both the Logistic Regression and Random Forest models achieved a sensitivity of 52.6% (Table 4).

For iatrogenic PTB < 34 weeks, all models performed well. The Random Forest model achieved the highest AUC of 0.806 (95% CI 0.692–0.920), followed closely by Logistic Regression with an AUC of 0.800 (95% CI 0.674–0.926). The Logistic Regression model had the highest sensitivity of 0.762 at its optimal threshold and maintained the highest sensitivity of 76.2% at a fixed specificity of 80% (Table 5).

### 3.5. Prediction of Spontaneous and Iatrogenic PTB < 32 Weeks

For the earliest gestational age cutoff of <32 weeks, the Random Forest model had the highest AUC for predicting spontaneous PTB (AUC 0.749, 95% CI 0.561–0.936). The Logistic Regression model recorded the highest sensitivity of 0.727 at its optimal threshold. At 80% specificity, the Random Forest model was the most sensitive, achieving a sensitivity of 63.6% (Table 6).

For iatrogenic PTB < 32 weeks, the Neural Network model was the top performer with an AUC of 0.862 (95% CI 0.772–0.953), followed by the Logistic Regression model (AUC 0.838, 95% CI 0.712–0.965). At a fixed specificity of 80%, the Random Forest, XGBoost, and Neural Network models all achieved a sensitivity of 75.0% (Table 7).

### 3.6. Comparative Model Performance for PTB < 37 Weeks

**Figure 1:**
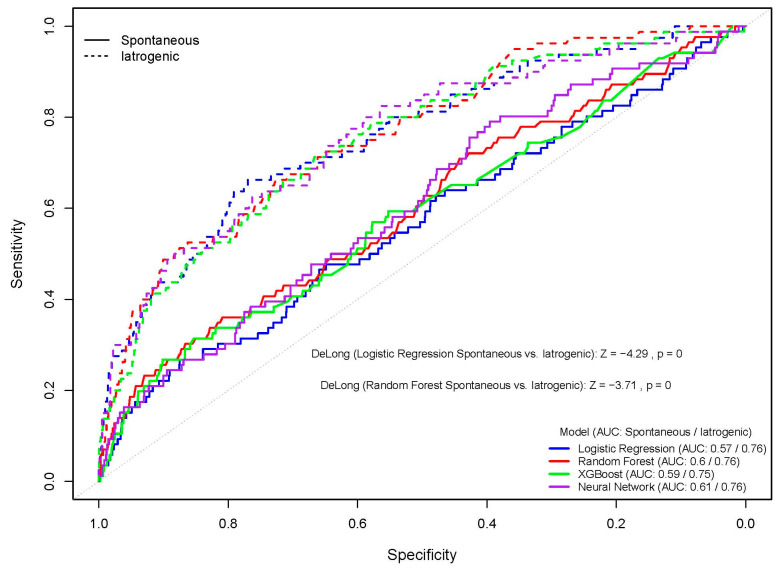
Receiver Operating Characteristic (ROC) curves for the models, comparing predictive performance for spontaneous versus iatrogenic PTB < 37 weeks.

The ROC curves for the models PTB < 37 weeks visually confirm the superior performance for iatrogenic PTB (dashed lines) compared to spontaneous PTB (solid lines) across all tested algorithms. Formal analysis using DeLong’s test confirmed this observation for both the Logistic Regression and Random Forest models. The AUC for Logistic Regression was significantly higher for iatrogenic PTB (AUC 0.76) than for spontaneous PTB (AUC 0.57) (Z = −4.29, *p* = 0). Similarly, the Random Forest model’s performance was significantly better for iatrogenic PTB (AUC 0.76) compared to spontaneous PTB (AUC 0.6) (Z = −3.71, *p* = 0) (Figure 1).

### 3.7. Model Interpretation with Shapley Values

To understand the decision-making process of the models for the <37 weeks PTB, SHAP analysis was used to identify the impact of each feature on the predictions of the Logistic Regression and Random Forest algorithms. This approach revealed distinct predictive factors for each PTB subtype and highlighted important differences between the modeling algorithms, enhancing the interpretability of their outputs.

**Figure 2:**
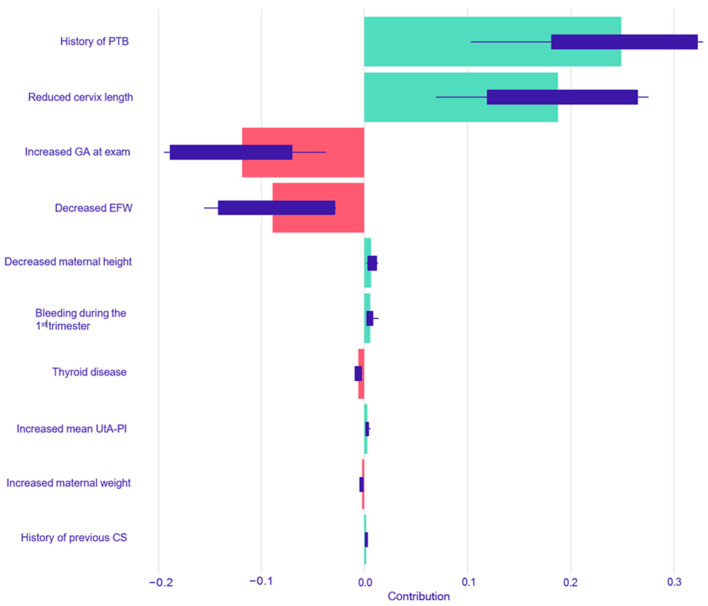
SHAP summary plot for the Logistic Regression model predicting spontaneous PTB < 37 weeks. The plot displays the top 10 most influential predictors, ranked by their mean absolute SHAP value. For each predictor, the horizontal bar represents the average impact on the model’s output. Green bars indicate a positive contribution (increasing the predicted risk of spontaneous PTB), while red bars indicate a negative contribution (decreasing the risk). Abbreviations: PTB, preterm birth; GA, gestational age; EFW, estimated fetal weight; UtA-PI, uterine artery pulsatility index; CS, cesarean section.

**Figure 3:**
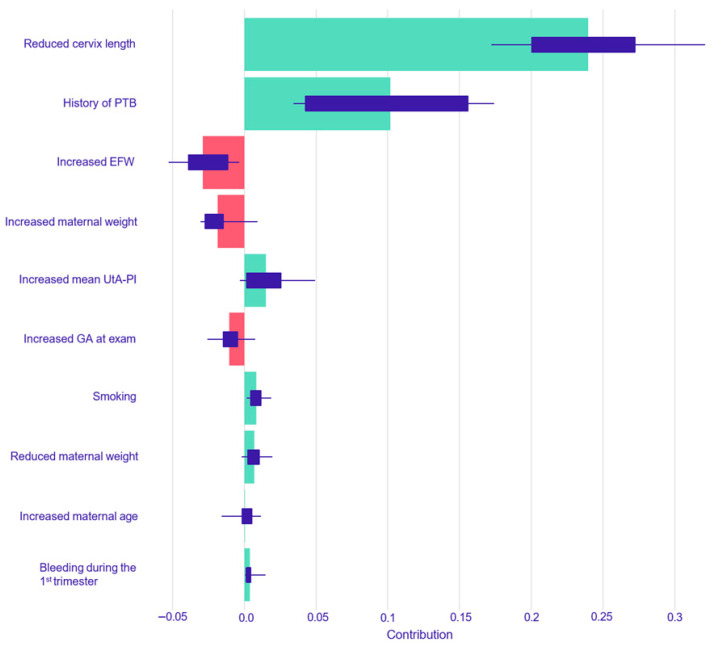
SHAP summary plot for the Random Forest model predicting spontaneous PTB < 37 weeks. The plot displays the top 10 most influential predictors for the Random Forest model, ranked by their mean absolute SHAP value. Green bars indicate a positive contribution (increasing the predicted risk of spontaneous PTB), while red bars indicate a negative contribution (decreasing the risk). Abbreviations: EFW, estimated fetal weight; UtA-PI, uterine artery pulsatility index; GA, gestational age.

For the prediction of spontaneous PTB < 37 weeks, SHAP analysis of the Logistic Regression model identified a history of PTB as the predictor with the largest positive impact on risk, followed by reduced cervical length. Increased gestational age at exam and decreased estimated fetal weight had the largest risk-reducing impacts (Figure 2). The Random Forest model for spontaneous PTB also identified reduced cervical length and a history of preterm delivery as the two most important risk-increasing factors. The most significant factors associated with a decreased risk in this model were an increased EFW and increased maternal weight (Figure 3).

**Figure 4:**
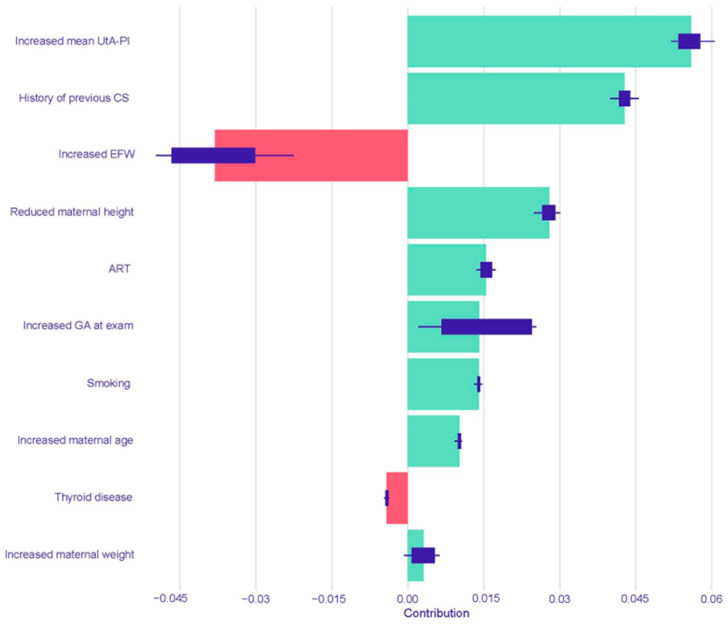
SHAP summary plot for the Logistic Regression model predicting iatrogenic PTB < 37 weeks. The plot displays the top 10 most influential predictors, ranked by their mean absolute SHAP value. Green bars indicate a positive contribution (increasing the predicted risk of iatrogenic PTB), while red bars indicate a negative contribution (decreasing the risk). Abbreviations: UtA-PI, uterine artery pulsatility index; CS, cesarean section; EFW, estimated fetal weight; ART, assisted reproductive technology; GA, gestational age.

**Figure 5:**
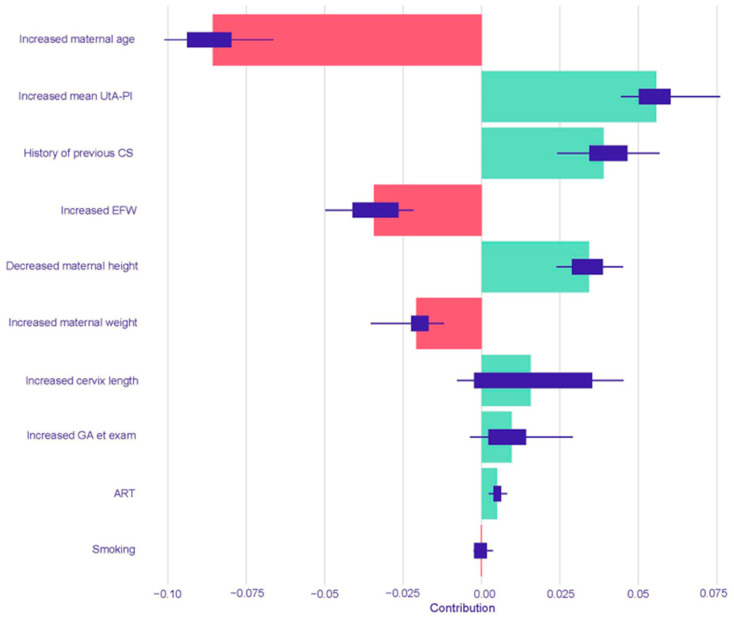
SHAP summary plot for the Random Forest model predicting iatrogenic PTB < 37 weeks. The plot displays the top 10 most influential predictors for the Random Forest model, ranked by their mean absolute SHAP value. Green bars indicate a positive contribution (increasing the predicted risk of iatrogenic PTB), while red bars indicate a negative contribution (decreasing the risk). Abbreviations: UtA-PI, uterine artery pulsatility index; CS, cesarean section; EFW, estimated fetal weight; GA, gestational age; ART, assisted reproductive technology.

For the prediction of iatrogenic PTB < 37 weeks, the Logistic Regression model identified an increased mean UtA-PI as the most influential risk-increasing predictor, followed by a history of a previous cesarean section. The factors with the largest risk-reducing impact were increased EFW and an increased gestational age at examination (Figure 4). In the Random Forest model for iatrogenic PTB, the leading risk-increasing factors were an increased mean UtA-PI, a history of a previous cesarean section, and decreased maternal height. Notably, increased maternal age was the most powerful risk-reducing predictor in this model, followed by increased EFW and increased maternal weight (Figure 5).

## 4. Discussion

### 4.1. Main Findings

This study yielded four principal findings that underscore the distinct nature of spontaneous and iatrogenic PTB. First, our predictive models demonstrated a significantly more robust and reliable performance in predicting iatrogenic PTB compared to spontaneous PTB across all gestational age thresholds. Second, this predictive accuracy was not static; for both subtypes, the models performed consistently better at identifying the risk for earlier and more clinically severe degrees of prematurity. A third key finding was the comparable performance between traditional Logistic Regression and the more complex machine learning models across most prediction tasks. Finally, our use of interpretable machine learning elucidated the divergent pathophysiological pathways driving these outcomes, providing clear insight into the models’ decision-making. The analysis showed that iatrogenic PTB was primarily driven by markers of placental dysfunction, while the prediction of spontaneous PTB was most influenced by a history of the condition and the presence of a short cervix.

### 4.2. Interpretation of Our Findings

This work is situated within a broad field of research for PTB prediction, which has reported a wide range of performance metrics [23]. For instance, recent machine learning models developed on large-scale data that combine PTB subtypes report strong predictive performances, with AUCs in the range of 0.73 to 0.75 [21,22]. Notably, our model focusing solely on iatrogenic PTB achieves a comparable AUC of 0.764, suggesting that the stronger predictive signals from indicated PTB may be the primary drivers of performance in those combined models. Therefore, a key distinction of our study is the systematic development and comparison of predictive models for iatrogenic and spontaneous PTB as separate endpoints. This approach addresses a significant gap in the literature by avoiding the common pitfall of grouping these distinct clinical entities. By doing so, we ensure that the unique contribution of each variable is accurately captured for each subtype, preventing a situation where a factor’s opposing effects on spontaneous versus iatrogenic PTB might cancel each other out. This demonstrates that an integrated, subtype-specific approach using routinely collected mid-gestation data is a valuable strategy for PTB prediction.

By effectively combining this readily available clinical information, we developed models with immediate clinical applicability that contribute to the emerging era of precision medicine. Our work demonstrates that a robust predictive framework can be achieved even with standard, interpretable models. Notably, all algorithms tested, from traditional Logistic Regression to more complex machine learning methods, yielded comparable predictive performance. This key finding underscores that the clinical value lies in the subtype-specific modeling strategy itself, which better isolates the distinct drivers of each outcome, rather than in the complexity of the algorithm used. A deeper analysis of these findings reveals critical insights, beginning with the marked difference in predictive power between the two PTB subtypes.

### 4.3. Differential Performance: Spontaneous vs. Iatrogenic Preterm Birth

A striking finding of our study is the significant disparity in model performance between the two primary subtypes of PTB. For PTB before 37 weeks, all models performed substantially better at predicting iatrogenic deliveries (AUCs up to 0.764) compared to spontaneous deliveries (AUCs up to 0.609). This difference was statistically significant and likely reflects the fundamental etiological distinctions between these two clinical entities. Our findings for spontaneous PTB, for delivery <37 weeks, align with the existing literature that underscores the challenge of its prediction. A systematic review and external validation by Meertens et al. found that even promising models for spontaneous PTB performed moderately, with validated AUCs ranging from 0.54 to 0.67 [23], a range within which our model’s performance (AUC 0.609) is consistent.

Spontaneous PTB is recognized as a complex syndrome with a multifactorial and often elusive etiology [33]. The difficulty in its prediction stems from significant pathophysiologic and genetic heterogeneities [25]. It is not a condition initiated by a single cause, but an overarching syndrome that can be triggered by diverse factors, which activate distinct but overlapping molecular pathways [33,34,35]. Furthermore, these disease processes can originate in any number of feto-maternal tissues, including the placenta, fetal membranes, or decidua, each responding uniquely to pro-parturition signals. This complex interplay makes it difficult to establish a universal predictive model, as a precise mechanism often cannot be identified in most individual cases [33,34,35]. Conversely, iatrogenic PTB is a medically indicated intervention, frequently prompted by maternal or fetal complications, and these conditions are often preceded by well-defined clinical and sonographic markers of placental insufficiency. Key indications, according to the American College of Obstetricians and Gynecologists guidelines, include hypertensive disorders, such as PE with severe features, and poorly controlled diabetes [36]. Fetal or placental indications often involve conditions like fetal growth restriction with abnormal umbilical artery Doppler flow, placenta previa, or vasa previa [36].

This is strongly corroborated by our model’s interpretability analysis using SHAP values. The SHAP analysis for iatrogenic PTB < 37 weeks heavily weighted markers of placental function and obstetric history. In both the Logistic Regression and Random Forest models, an increased mean UtA-PI, a direct measure of placental vascular resistance, was a leading predictor for an increased risk of iatrogenic PTB. Abnormal uterine artery Doppler velocimetry is a well-established indicator of impaired placentation and is strongly associated with adverse pregnancy outcomes like pre-eclampsia and fetal growth restriction, which are leading causes of indicated PTB [10]. A history of a previous cesarean section was another powerful risk-increasing factor in both models. This finding aligns with its established role as a risk factor for subsequent placental abnormalities and as a marker for prior obstetric complications, which carry a significant risk of recurrence [37].

In contrast, the prediction of spontaneous PTB < 37 weeks was predominantly driven by factors reflecting a predisposition to preterm labor and cervical incompetence. The SHAP analyses confirmed this, identifying a history of PTB and a reduced cervical length as the most powerful predictors of spontaneous PTB across both models. A history of PTB is a well-documented and significant risk factor, while cervical length is a cornerstone of modern PTB screening [38]. A shortened cervix is a clear biophysical marker of the premature initiation of the parturition process, and its high importance in the models, particularly the Random Forest model, where it was the top feature, underscores its clinical significance [38].

A noteworthy finding from the SHAP analysis is the differing interpretation of key variables between the Logistic Regression and Random Forest models, particularly for iatrogenic PTB. For instance, while the Logistic Regression model identified increased maternal age as a risk-increasing factor, the Random Forest model found it to be the most powerful predictor for decreasing risk. This apparent contradiction likely stems from the fundamental differences between the algorithms. Logistic Regression models linear relationships, assessing the average effect of each variable independently. In contrast, Random Forest, a tree-based ensemble, can capture complex, non-linear interactions. The model may have learned that while older age is correlated with certain risk factors, in the absence of those factors (e.g., no history of CS, normal UtA-PI), it is associated with a lower risk of iatrogenic intervention. This ability to model such nuanced, conditional relationships highlights a key advantage of non-linear models in capturing the intricate interplay of clinical variables.

### 4.4. Performance Across Gestational Age Cutoffs

Another important observation was the consistent improvement in model performance for earlier gestational age cutoffs. This trend was particularly pronounced for iatrogenic PTB, where the predictive accuracy increased substantially with the severity of prematurity. The best-performing model for iatrogenic delivery <37 weeks achieved an AUC of 0.764 (Random Forest), which rose to 0.806 for delivery <34 weeks (Random Forest), and peaked at an AUC of 0.862 for delivery <32 weeks (Neural Network) (Table 3, Table 5, and Table 7). This strong positive correlation between predictive power and degree of prematurity suggests that the underlying pathological processes leading to very early indicated deliveries are more pronounced and, therefore, more readily detectable by the variables in our models. Severe, early-onset placental dysfunction, for example, is likely to manifest with more extreme deviations in uterine artery Doppler indices and fetal growth parameters, thereby providing a stronger predictive signal.

A similar, albeit less dramatic, trend was observed for spontaneous PTB. The predictive accuracy for spontaneous delivery <37 weeks was modest, with the best model achieving an AUC of 0.609 (Neural Network). However, performance improved to an AUC of 0.678 for delivery <34 weeks (Random Forest) and reached an AUC of 0.749 for the <32 weeks outcome (Random Forest). This progression suggests that while late spontaneous PTBs are difficult to distinguish from term births using mid-gestation data, the most severe cases of spontaneous PTB are better captured by our model’s variables. It is in these earliest predictions that machine learning models appeared to offer a slight advantage. For instance, in predicting spontaneous PTB < 32 weeks, the Random Forest model achieved a higher AUC (0.749) than Logistic Regression (0.685). This may suggest that more complex algorithms have a potential edge in capturing the non-linear patterns and complex associations that are more pronounced in extreme-risk pregnancies, where predictive precision is most vital.

This finding is also supported by previous studies in the area, which show that prediction increases as the gestational age threshold decreases [12,23]. The improved accuracy for the earliest and most clinically significant deliveries is a key strength of our models, as these are the cases associated with the highest rates of neonatal morbidity and mortality. This is crucial because the risks for adverse outcomes directly increase with the degree of prematurity [10]. Globally, approximately 15% of all preterm births, around 2 million babies in 2020, occurred before 32 weeks of gestation and required more intensive neonatal care [2]. Better prediction for these earliest deliveries is paramount, given that direct complications from PTB were the leading cause of child mortality in 2019 [2].

### 4.5. Clinical Implications

The development of robust, accessible, and subtype-specific predictive models has significant clinical implications. By integrating multiple data points, these models provide a personalized risk assessment that surpasses the limitations of relying on single risk factors. This distinction is crucial, as the management strategies for women at high risk for each subtype differ substantially. A high predicted risk for spontaneous PTB may prompt interventions like progesterone therapy or cervical cerclage [13], while a high risk for iatrogenic PTB necessitates intensified surveillance for conditions like PE and FGR.

Furthermore, accurate risk stratification is essential for optimizing perinatal outcomes. Identifying women at high risk for PTB allows for the timely administration of antenatal corticosteroids to promote fetal lung maturity and magnesium sulfate for neuroprotection, interventions proven to reduce neonatal morbidity [16,17]. This early risk stratification prompts intensified clinical surveillance, which in turn ensures that these time-sensitive treatments are administered without delay when the clinical signs of imminent delivery emerge. Beyond individual patient care, these predictive tools have important logistical benefits [18]. Reliable prediction can aid in healthcare resource planning, ensuring the availability of neonatal intensive care unit beds and specialized staff, and facilitating the timely transfer of at-risk mothers to tertiary care centers [18]. Our models are built on a set of non-invasive variables already collected during routine prenatal care. This provides a foundation for the potential development of easy-to-use, low-cost clinical decision support tools. If externally validated, such tools could be integrated into everyday practice to help refine risk stratification and support a more personalized approach to perinatal medicine.

### 4.6. Strengths and Limitations

The primary strengths of this study include its novel approach of developing and validating separate predictive models for spontaneous and iatrogenic PTB, its large cohort size, the use of prospectively collected data within a standardized academic protocol, and a robust methodological approach comparing several algorithms, including both traditional regression and machine learning methods, with rigorous internal validation. Furthermore, the use of SHAP for model interpretation provides valuable insights into the clinical drivers of prediction, enhancing the transparency and trustworthiness of the models.

However, the study has several limitations. The retrospective design, although based on prospectively collected data, is one such limitation. The single-center nature of the study may affect the generalizability of our findings to other populations. A significant limitation is the potential intervention bias. In our cohort, women at high risk for pre-eclampsia received aspirin, and those with a short cervix were treated with progesterone. These interventions may have prevented or delayed some PTBs, potentially leading to an underestimation of our models’ predictive performance in an untreated population. Finally, while our models were robustly validated internally, they have not yet undergone external validation in an independent dataset. This is a significant limitation, as the generalizability of the findings is uncertain until performance is confirmed in different clinical populations. External validation is a crucial next step before widespread clinical implementation can be recommended.

## 5. Conclusions

In conclusion, our study demonstrates that routine mid-gestation data may effectively predict iatrogenic PTB, with accuracy improving for earlier gestations. In contrast, the prediction of spontaneous PTB was modest, suggesting that future models may require additional data, such as biochemical markers. A key finding was that traditional Logistic Regression performed comparably to complex machine learning algorithms. This highlights that the clinical value is rooted in our subtype-specific approach, which isolates distinct drivers for each outcome, rather than in algorithmic complexity. Future research must prioritize the external validation of these models to confirm their clinical utility and generalizability.

## Figures and Tables

**Table 1:** Population characteristics.

Variable	Level	PTB < 37 (*n* = 857)	Term ≥ 37 (*n* = 8948)	*p*-Value	Missing (%)
**Maternal**					
Maternal Age (mean (SD))		32.28 (5.84)	31.62 (5.23)	<0.001	0
Height (cm) (mean (SD))		164.64 (6.14)	165.66 (5.98)	<0.001	6
Pre-pregnancy Weight (kg) (median [IQR])		63.00 [56.38, 78.00]	63.00 [57.00, 73.00]	0.216	10.7
Pre-pregnancy BMI (median [IQR])		23.40 [20.90, 28.40]	23.00 [20.80, 26.30]	0.001	10.8
Multiparous (%)		363 (42.4)	3722 (41.6)	0.692	0
Current Smoker (%)		113 (13.2)	947 (10.6)	0.022	0
Medical and Obstetric History					
Assisted Reproductive Technology (%)		78 (9.1)	450 (5.0)	<0.001	0
Aspirin Use (%)		155 (18.1)	903 (10.1)	<0.001	0
Cervical Cerclage(%)		6 (0.7)	23 (0.3)	0.051	0
First Trimester Bleeding (%)		110 (12.8)	651 (7.3)	<0.001	0
Preeclampsia (%)		38 (4.4)	30 (0.3)	<0.001	0
Gestational Diabetes (%)		134 (15.6)	1158 (12.9)	0.03	0
Family History of Diabetes (%)		0 (0.0)	12 (0.1)	0.575	0
History of Fetal Death (%)		11 (1.3)	27 (0.3)	<0.001	0
History of LOOP/LLETZ Procedure (%)		6 (0.7)	19 (0.2)	0.019	0
History of Preterm Delivery (%)		87 (10.2)	246 (2.7)	<0.001	0
Nulliparity (%)		501 (58.5)	5254 (58.7)	0.88	0
Previous Cesarean Section (%)		188 (21.9)	1466 (16.4)	<0.001	3.4
Pre-existing Diabetes (%)		12 (1.4)	30 (0.3)	<0.001	0
Chronic Hypertension (%)		16 (1.9)	19 (0.2)	<0.001	0
Epilepsy (%)		2 (0.2)	18 (0.2)	1	0
Thrombophilia (%)		20 (2.3)	126 (1.4)	0.047	0
Thyroid Disorder (%)		75 (8.8)	698 (7.8)	0.357	0
**Ultrasound and Fetal Markers**					
Gestational Age at Exam (weeks) (mean (SD))		22.22 (0.77)	22.17 (0.65)	0.052	0
Biparietal Diameter (mm) (mean (SD))		53.81 (2.78)	54.02 (2.60)	0.033	1.2
Abdominal Circumference (mm) (mean (SD))		172.35 (10.47)	173.73 (9.00)	<0.001	1.2
Femur Length (mm) (mean (SD))		37.12 (2.42)	37.64 (2.12)	<0.001	1.2
Estimated Fetal Weight (median [IQR])		468.50 [433.00, 511.00]	480.00 [444.00, 518.00]	<0.001	1.3
Estimated Fetal Weight percentile (median [IQR])		52.42 [26.10, 78.43]	62.12 [38.46, 82.28]	<0.001	1.3
Estimated Fetal Weight Z-Score (mean (SD))		−0.04 (1.45)	0.32 (0.90)	<0.001	1.3
Nuchal Fold Thickness (mm) (mean (SD))		4.20 (0.63)	4.32 (0.61)	<0.001	2.2
Cisterna Magna Diameter (mm) (mean (SD))		5.56 (0.82)	5.70 (0.80)	<0.001	2.6
Transcerebellar Diameter (mm) (mean (SD))		23.49 (1.14)	23.57 (1.09)	0.035	2.1
Posterior Ventricle Diameter (mm) (median [IQR])		6.40 [5.90, 6.80]	6.30 [5.80, 6.90]	0.587	2.4
Cervical Length (mm) (median [IQR])		33.00 [30.00, 36.00]	34.00 [31.00, 37.00]	<0.001	2
Mean Uterine Artery PI (median [IQR])		1.09 [0.88, 1.39]	0.98 [0.84, 1.16]	<0.001	1.5
Uterine Artery PI Centile (median [IQR])		64.53 [28.22, 92.04]	46.09 [21.90, 73.37]	<0.001	1.5
Uterine Artery PI Z-Score (mean (SD))		0.51 (1.41)	−0.03 (1.04)	<0.001	1.5
Fetal Presentation (%)	Cephalic	490 (57.2)	4831 (54.0)	<0.001	1.3
	Breech	300 (35.0)	3636 (40.6)		
	Transverse	35 (4.1)	384 (4.3)		
Fetal Sex (%)	Female	384 (44.8)	4206 (47.0)	<0.001	2.7
	Male	416 (48.5)	4535 (50.7)		
Cervical Funneling (%)		43 (5.0)	131 (1.5)	<0.001	2
Placenta Location (%)	High	694 (81.0)	7566 (84.6)	<0.001	1.4
	Low	103 (12.0)	1213 (13.6)		
	Previa	29 (3.4)	66 (0.7)		
Bilobate Placenta (%)		38 (4.4)	190 (2.1)	<0.001	1.4
Placenta Grade (%)	Grade 1	19 (2.2)	81 (0.9)	<0.001	1.1
	Grade 2	12 (1.4)	9 (0.1)		
Single Umbilical Artery (%)		17 (2.0)	96 (1.1)	<0.001	1.8
Cord Insertion (%)	central	165 (19.3)	2230 (24.9)	<0.001	9.6
	eccentric	436 (50.9)	4922 (55.0)		
	marginal	111 (13.0)	872 (9.7)		
	velamentous	32 (3.7)	91 (1.0)		
Vasa Previa (%)		8 (0.9)	2 (0.0)	<0.001	0
Uterine Artery Notch (%)	Unilateral	71 (8.3)	389 (4.3)	<0.001	1.5
	Bilateral	54 (6.3)	92 (1.0)		
**Delivery Outcomes**					
Onset of Preterm Labor, *n* (%)	Iatrogenic	402 (46.9)	Not applicable	<0.001	7
	Spontaneous	433 (50.5)	Not applicable		
Birth Weight (g) (median [IQR])		2400 [1960, 2830]	3280 [3030, 3570]	<0.001	0.9
Gestational Age at Delivery (w) (median [IQR])		35.57 [33.86, 36.43]	39.14 [38.43, 39.86]	<0.001	0
Delivery Outcome (%)	Fetal Death	37 (4.3)	11 (0.1)	<0.001	0
	Neonatal Death < 1 w	17 (2.0)	7 (0.1)		
	Neonatal Death > 1 w	4 (0.5)	3 (0.0)		
	Live Birth	799 (93.2)	8927 (99.8)		

Continuous variables are presented as mean (standard deviation, SD) or median [interquartile range, IQR]. Statistical comparisons were made using Student’s *t*-test for normally distributed variables, Mann–Whitney U test for non-normally distributed variables, and the Chi-squared or Fisher’s exact test for categorical variables. Abbreviations: LOOP/LLETZ, loop electrosurgical excision procedure/large loop excision of the transformation zone; PI, pulsatility index.

**Table 2:** Performance of the models for the prediction of spontaneous PTB < 37 weeks.

Model	AUC (95% CI)	Accuracy	Sensitivity	Specificity	PPV	F1-Score	Sensitivity at 80% Specificity
Logistic Regression	0.567 (0.500–0.634)	0.695	0.372	0.710	0.058	0.101	30.2%
Random Forest	0.603 (0.538–0.669)	0.749	0.360	0.767	0.069	0.116	36.0%
XGBoost	0.586 (0.520–0.653)	0.678	0.407	0.691	0.060	0.104	33.7%
Neural Network	0.609 (0.546–0.671)	0.687	0.430	0.700	0.064	0.112	30.2%

Performance metrics calculated on the 20% hold-out test set. Abbreviations: AUC, Area Under the Curve; CI, Confidence Interval; PPV, Positive Predictive Value.

**Table 3:** Performance of the models for the prediction of iatrogenic PTB < 37 weeks.

Model	AUC (95% CI)	Accuracy	Sensitivity	Specificity	PPV	F1-Score	Sensitivity at 80% Specificity
Logistic Regression	0.760 (0.703–0.817)	0.722	0.675	0.724	0.099	0.172	60.0%
Random Forest	0.764 (0.710–0.818)	0.785	0.537	0.796	0.105	0.176	53.7%
XGBoost	0.754 (0.698–0.810)	0.732	0.613	0.738	0.095	0.164	52.5%
Neural Network	0.761 (0.703–0.819)	0.787	0.550	0.798	0.108	0.181	55.0%

Performance metrics calculated on the 20% hold-out test set. Abbreviations: AUC, Area Under the Curve; CI, Confidence Interval; PPV, Positive Predictive Value.

**Table 4:** Performance of the models for the prediction of spontaneous PTB < 34 weeks.

Model	AUC (95% CI)	Accuracy	Sensitivity	Specificity	PPV	F1-Score	Sensitivity at 80% Specificity
Logistic Regression	0.624 (0.445–0.804)	0.680	0.579	0.681	0.018	0.034	52.6%
Random Forest	0.678 (0.525–0.831)	0.747	0.526	0.749	0.020	0.039	52.6%
XGBoost	0.637 (0.497–0.777)	0.675	0.474	0.677	0.014	0.028	36.8%
Neural Network	0.671 (0.519–0.823)	0.775	0.474	0.778	0.021	0.040	47.4%

Performance metrics calculated on the 20% hold-out test set. Abbreviations: AUC, Area Under the Curve; CI, Confidence Interval; PPV, Positive Predictive Value.

**Table 5:** Performance of the models for the prediction of iatrogenic PTB < 34 weeks.

Model	AUC (95% CI)	Accuracy	Sensitivity	Specificity	PPV	F1-Score	Sensitivity at 80% Specificity
Logistic Regression	0.800 (0.674–0.926)	0.793	0.762	0.793	0.039	0.074	76.2%
Random Forest	0.806 (0.692–0.920)	0.804	0.714	0.805	0.039	0.073	71.4%
XGBoost	0.717 (0.575–0.860)	0.670	0.667	0.670	0.022	0.042	52.4%
Neural Network	0.752 (0.631–0.873)	0.705	0.714	0.705	0.026	0.050	61.9%

Performance metrics calculated on the 20% hold-out test set. Abbreviations: AUC, Area Under the Curve; CI, Confidence Interval; PPV, Positive Predictive Value.

**Table 6:** Performance of the models for the prediction of spontaneous PTB < 32 weeks.

Model	AUC (95% CI)	Accuracy	Sensitivity	Specificity	PPV	F1-Score	Sensitivity at 80% Specificity
Logistic Regression	0.685 (0.466–0.904)	0.626	0.727	0.625	0.011	0.022	54.5%
Random Forest	0.749 (0.561–0.936)	0.862	0.455	0.864	0.019	0.036	63.6%
XGBoost	0.628 (0.397–0.858)	0.555	0.636	0.555	0.008	0.016	54.5%
Neural Network	0.675 (0.493–0.857)	0.795	0.545	0.796	0.015	0.029	54.5%

Performance metrics calculated on the 20% hold-out test set. Abbreviations: AUC, Area Under the Curve; CI, Confidence Interval; PPV, Positive Predictive Value.

**Table 7:** Performance of the models for the prediction of iatrogenic PTB < 32 weeks.

Model	AUC (95% CI)	Accuracy	Sensitivity	Specificity	PPV	F1-Score	Sensitivity at 80% Specificity
Logistic Regression	0.838 (0.712–0.965)	0.760	0.750	0.760	0.019	0.037	66.7%
Random Forest	0.804 (0.629–0.979)	0.870	0.667	0.871	0.031	0.059	75.0%
XGBoost	0.793 (0.605–0.981)	0.753	0.750	0.753	0.019	0.036	75.0%
Neural Network	0.862 (0.772–0.953)	0.816	0.750	0.816	0.025	0.048	75.0%

Performance metrics calculated on the 20% hold-out test set. Abbreviations: AUC, Area Under the Curve; CI, Confidence Interval; PPV, Positive Predictive Value.

## Data Availability

Data available upon reasonable request due to privacy reasons.

## Abbreviations

The following abbreviations are used in this manuscript:

ART	Assisted Reproductive Technology
AUC	Area Under the Curve
CI	Confidence Interval
CS	Cesarean Section
EFW	Estimated Fetal Weight
GA	Gestational Age
ML	Machine Learning
PPV	Positive Predictive Value
PTB	Preterm Birth
ROC	Receiver Operating Characteristic
SHAP	SHapley Additive exPlanations
UtA-PI	Uterine Artery Pulsatility Index

## References

[1] (1977). WHO: Recommended definitions, terminology and format for statistical tables related to the perinatal period and use of a new certificate for cause of perinatal deaths. Modifications recommended by FIGO as amended October 14, 1976. Acta Obstet. Gynecol. Scand..

[2] Ohuma E.O., Moller A.-B., Bradley E., Chakwera S., Hussain-Alkhateeb L., Lewin A., Okwaraji Y.B., Mahanani W.R., Johansson E.W., Lavin T. (2023). National, regional, and global estimates of preterm birth in 2020, with trends from 2010: A systematic analysis. Lancet.

[3] Goldenberg R.L. (2002). The management of preterm labor. Obstet. Gynecol..

[4] Jelliffe-Pawlowski L.L., Baer R.J., Oltman S., McKenzie-Sampson S., Afulani P., Amsalu R., Bell A.J., Blebu B., Blackman K.C.A., Chambers C.D. (2024). Risk and Protective Factors for Preterm Birth Among Racial, Ethnic, and Socioeconomic Groups in California. JAMA Netw. Open.

[5] Blencowe H., Cousens S., Chou D., Oestergaard M., Say L., Moller A.B., Kinney M., Lawn J. (2013). Born too soon: The global epidemiology of 15 million preterm births. Reprod. Health.

[6] Liu T., Xu Y., Gong Y., Zheng J., Chen Z. (2024). The global burden of disease attributable to preterm birth and low birth weight in 204 countries and territories from 1990 to 2019: An analysis of the Global Burden of Disease Study. J. Glob. Health.

[7] Siffel C., Hirst A.K., Sarda S.P., Chen H., Ferber J., Kuzniewicz M.W., Li D.K. (2022). The clinical burden of extremely preterm birth in a large medical records database in the United States: Complications, medication use, and healthcare resource utilization. J. Matern. Fetal Neonatal Med..

[8] Keiser A.M., Schmidt B. (2025). Lasting Burden of Preterm Birth on Health and Health Services. JAMA Pediatr..

[9] Campbell S. (2018). Prevention of spontaneous preterm birth: Universal cervical length assessment and vaginal progesterone in women with a short cervix: Time for action!. Am. J. Obstet. Gynecol..

[10] Papastefanou I., Gyokova E., Gungil B., Syngelaki A., Nicolaides K.H. (2023). Prediction of adverse perinatal outcome at midgestation. Ultrasound Obstet. Gynecol..

[11] Johnston K.M., Gooch K., Korol E., Vo P., Eyawo O., Bradt P., Levy A. (2014). The economic burden of prematurity in Canada. BMC Pediatr..

[12] Souka A.P., Papastefanou I., Pilalis A., Kassanos D., Papadopoulos G. (2019). Implementation of universal screening for preterm delivery by mid-trimester cervical-length measurement. Ultrasound Obstet. Gynecol..

[13] Dagklis T., Akolekar R., Villalain C., Tsakiridis I., Kesrouani A., Tekay A., Plasencia W., Wellmann S., Kusuda S., Jekova N. (2023). Management of preterm labor: Clinical practice guideline and recommendation by the WAPM-World Association of Perinatal Medicine and the PMF-Perinatal Medicine Foundation. Eur. J. Obstet. Gynecol. Reprod. Biol..

[14] Romero R., Conde-Agudelo A., Da Fonseca E., O’Brien J.M., Cetingoz E., Creasy G.W., Hassan S.S., Nicolaides K.H. (2018). Vaginal progesterone for preventing preterm birth and adverse perinatal outcomes in singleton gestations with a short cervix: A meta-analysis of individual patient data. Am. J. Obstet. Gynecol..

[15] Conde-Agudelo A., Romero R., Da Fonseca E., O’Brien J.M., Cetingoz E., Creasy G.W., Hassan S.S., Erez O., Pacora P., Nicolaides K.H. (2018). Vaginal progesterone is as effective as cervical cerclage to prevent preterm birth in women with a singleton gestation, previous spontaneous preterm birth, and a short cervix: Updated indirect comparison meta-analysis. Am. J. Obstet. Gynecol..

[16] Sweet D.G., Carnielli V., Greisen G., Hallman M., Ozek E., Te Pas A., Plavka R., Roehr C.C., Saugstad O.D., Simeoni U. (2019). European Consensus Guidelines on the Management of Respiratory Distress Syndrome—2019 Update. Neonatology.

[17] Doyle L.W., Crowther C.A., Middleton P., Marret S., Rouse D. (2009). Magnesium sulphate for women at risk of preterm birth for neuroprotection of the fetus. Cochrane Database Syst. Rev..

[18] Desplanches T., Morgan A.S., Jones P., Diguisto C., Zeitlin J., Martin-Marchand L., Benhammou V., Lecomte B., Rozé J.C., Truffert P. (2021). Risk factors for very preterm delivery out of a level III maternity unit: The EPIPAGE-2 cohort study. Paediatr. Perinat. Epidemiol..

[19] Papastefanou I., Wright D., Syngelaki A., Akolekar R., Nicolaides K.H. (2023). Personalized stratification of pregnancy care for small for gestational age neonates from biophysical markers at midgestation. Am. J. Obstet. Gynecol..

[20] Nicolaides K.H., Papastefanou I., Syngelaki A., Ashoor G., Akolekar R. (2022). Predictive performance for placental dysfunction related stillbirth of the competing risks model for small-for-gestational-age fetuses. BJOG.

[21] Ebrahimvandi A., Hosseinichimeh N., Kong Z.J. (2022). Identifying the Early Signs of Preterm Birth from U.S. Birth Records Using Machine Learning Techniques. Information.

[22] Khan W., Zaki N., Ghenimi N., Ahmad A., Bian J., Masud M.M., Ali N., Govender R., Ahmed L.A. (2023). Predicting preterm birth using explainable machine learning in a prospective cohort of nulliparous and multiparous pregnant women. PLoS ONE.

[23] Meertens L.J.E., van Montfort P., Scheepers H.C.J., van Kuijk S.M.J., Aardenburg R., Langenveld J., van Dooren I.M.A., Zwaan I.M., Spaanderman M.E.A., Smits L.J.M. (2018). Prediction models for the risk of spontaneous preterm birth based on maternal characteristics: A systematic review and independent external validation. Acta Obstet. Gynecol. Scand..

[24] Fonseca E., Yu C.K.H., Singh M., Papageorghiou A.T., Nicolaides K.H. (2006). Relationship between second-trimester uterine artery Doppler and spontaneous early preterm delivery. Ultrasound Obstet. Gynecol..

[25] Włodarczyk T., Płotka S., Szczepański T., Rokita P., Sochacki-Wójcicka N., Wójcicki J., Lipa M., Trzciński T. (2021). Machine Learning Methods for Preterm Birth Prediction: A Review. Electronics.

[26] Akazawa M., Hashimoto K. (2022). Prediction of preterm birth using artificial intelligence: A systematic review. J. Obstet. Gynaecol..

[27] Zhang L., Li H., Li J., Hou Y., Xu B., Li N., Yang T., Liu C., Qiao C. (2020). Prediction of iatrogenic preterm birth in patients with scarred uterus: A retrospective cohort study in Northeast China. BMC Pregnancy Childbirth.

[28] Cuschieri S. (2019). The STROBE guidelines. Saudi J. Anaesth..

[29] Collins G.S., Reitsma J.B., Altman D.G., Moons K.G.M. (2015). Transparent reporting of a multivariable prediction model for individual prognosis or diagnosis (TRIPOD): The TRIPOD Statement. BMC Med..

[30] Hadlock F.P., Harrist R.B., Sharman R.S., Deter R.L., Park S.K. (1985). Estimation of fetal weight with the use of head, body, and femur measurements—A prospective study. Am. J. Obstet. Gynecol..

[31] Bhide A., Acharya G., Baschat A., Bilardo C.M., Brezinka C., Cafici D., Ebbing C., Hernandez-Andrade E., Kalache K., Kingdom J. (2021). ISUOG Practice Guidelines (updated): Use of Doppler velocimetry in obstetrics. Ultrasound Obstet. Gynecol..

[32] Shapley L.S. (1953). A Value for n-person Games. Contributions to the Theory of Games.

[33] Goldenberg R.L., Culhane J.F., Iams J.D., Romero R. (2008). Epidemiology and causes of preterm birth. Lancet.

[34] Menon R. (2008). Spontaneous preterm birth, a clinical dilemma: Etiologic, pathophysiologic and genetic heterogeneities and racial disparity. Acta Obstet. Gynecol. Scand..

[35] Vidal M.S., Lintao R.C.V., Severino M.E.L., Tantengco O.A.G., Menon R. (2022). Spontaneous preterm birth: Involvement of multiple feto-maternal tissues and organ systems, differing mechanisms, and pathways. Front. Endocrinol..

[36] (2021). Medically Indicated Late-Preterm and Early-Term Deliveries: ACOG Committee Opinion, Number 831. Obstet. Gynecol..

[37] Keag O.E., Norman J.E., Stock S.J. (2018). Long-term risks and benefits associated with cesarean delivery for mother, baby, and subsequent pregnancies: Systematic review and meta-analysis. PLoS Med..

[38] Coutinho C.M., Sotiriadis A., Odibo A., Khalil A., D’Antonio F., Feltovich H., Salomon L.J., Sheehan P., Napolitano R., Berghella V. (2022). ISUOG Practice Guidelines: Role of ultrasound in the prediction of spontaneous preterm birth. Ultrasound Obstet. Gynecol..

[39] Wade D.T. (2005). Ethics, audit, and research: All shades of grey. BMJ.

